# *In vitro* and *in vivo* effects of zoledronic acid on senescence and senescence-associated secretory phenotype markers

**DOI:** 10.18632/aging.204701

**Published:** 2023-05-07

**Authors:** Parinya Samakkarnthai, Dominik Saul, Lei Zhang, Zaira Aversa, Madison L. Doolittle, Jad G. Sfeir, Japneet Kaur, Elizabeth J. Atkinson, James R. Edwards, Graham G. Russell, Robert J. Pignolo, James L. Kirkland, Tamar Tchkonia, Laura J. Niedernhofer, David G. Monroe, Nathan K. Lebrasseur, Joshua N. Farr, Paul D. Robbins, Sundeep Khosla

**Affiliations:** 1 Division of Endocrinology, Mayo Clinic, Rochester, MN 55905, USA; 2 Robert and Arlene Kogod Center on Aging, Mayo Clinic, Rochester, MN 55905, USA; 3 Division of Endocrinology, Phramongkutklao Hospital and College of Medicine, Bangkok 10400, Thailand; 4 Department of Trauma and Reconstructive Surgery, Eberhard Karls University Tübingen, BG Trauma Center Tübingen, Tübingen 72076, Germany; 5 Institute on the Biology of Aging and Metabolism, Department of Biochemistry, Molecular Biology and Biophysics, University of Minnesota, Minneapolis, MN 55455, USA; 6 Department of Physical Medicine and Rehabilitation, Mayo Clinic, Rochester, MN 55905, USA; 7 Department of Quantitative Health Sciences, Mayo Clinic, Rochester, MN 55905, USA; 8 Botnar Research Centre, Nuffield Department of Orthopaedics, Rheumatology and Musculoskeletal Sciences, University of Oxford, Oxford, OX3 7FY, UK; 9 Mellanby Centre for Musculoskeletal Research, University of Sheffield, Sheffield, S10 2RX, UK; 10 Division of Hospital Internal Medicine, Mayo Clinic, Rochester, MN 55905, USA; 11 Department of Physiology and Biomedical Engineering, Mayo Clinic, Rochester, MN 55905, USA; 12 Division of General Internal Medicine, Mayo Clinic, Rochester, MN 55905, USA

**Keywords:** senescence, senolytics, aging, bone, bisphosphonates

## Abstract

In addition to reducing fracture risk, zoledronic acid has been found in some studies to decrease mortality in humans and extend lifespan and healthspan in animals. Because senescent cells accumulate with aging and contribute to multiple co-morbidities, the non-skeletal actions of zoledronic acid could be due to senolytic (killing of senescent cells) or senomorphic (inhibition of the secretion of the senescence-associated secretory phenotype [SASP]) actions. To test this, we first performed *in vitro* senescence assays using human lung fibroblasts and DNA repair-deficient mouse embryonic fibroblasts, which demonstrated that zoledronic acid killed senescent cells with minimal effects on non-senescent cells. Next, in aged mice treated with zoledronic acid or vehicle for 8 weeks, zoledronic acid significantly reduced circulating SASP factors, including CCL7, IL-1β, TNFRSF1A, and TGFβ1 and improved grip strength. Analysis of publicly available RNAseq data from CD115+ (CSF1R/c-fms+) pre-osteoclastic cells isolated from mice treated with zoledronic acid demonstrated a significant downregulation of senescence/SASP genes (SenMayo). To establish that these cells are potential senolytic/senomorphic targets of zoledronic acid, we used single cell proteomic analysis (cytometry by time of flight [CyTOF]) and demonstrated that zoledronic acid significantly reduced the number of pre-osteoclastic (CD115+/CD3e-/Ly6G-/CD45R-) cells and decreased protein levels of p16, p21, and SASP markers in these cells without affecting other immune cell populations. Collectively, our findings demonstrate that zoledronic acid has senolytic effects *in vitro* and modulates senescence/SASP biomarkers *in vivo*. These data point to the need for additional studies testing zoledronic acid and/or other bisphosphonate derivatives for senotherapeutic efficacy.

## INTRODUCTION

Aging is a major risk factor for multiple co-morbidities, including sarcopenia [1, 2], cardiovascular disease [3], cognitive disorders [4], arthritis [5], respiratory disorders [6], osteoporosis [7], and frailty [8]. Although treatments exist to individually alleviate the symptoms and/or progression of each of these diseases, with an increasing elderly population, the overall burden of these diseases is expected to increase markedly. Indeed, the global population aged 60 years and older is expected to increase from 8.5% to 16.7% of the population by 2050, thereby outnumbering adolescents and young individuals combined (aged 10-24 years) [9]. With this expectation of such a large demographic of aged individuals, the need to study, understand, and treat age-related disorders is more important than ever.

It has been postulated that targeting the fundamental mechanisms of aging may, in fact, delay or alleviate most age-related diseases. A well-studied contributor to aging is the age-related onset of cellular senescence, which is a state of growth arrest that occurs due to the accumulation of DNA damage and cellular stress [7, 10]. This is distinct from quiescence, as senescent cells are excluded from a reversible G0 state [11] and also acquire the senescence-associated secretory phenotype (SASP), which is characterized by the release of pro-inflammatory factors that have detrimental effects both locally and systemically on tissue function [7]. In addition, these SASP factors can further induce senescence in other cells [12, 13]. Chronic accumulation of senescent cells has been linked to common age-related diseases [10], and clearance of senescent cells in mice either through pharmacological or genetic methods extends healthspan [13, 14]. Further, senescent cell clearance in mice also alleviates the progression of systemic diseases, including cardiovascular disease [15, 16], physical frailty [13], pulmonary fibrotic disease [17], cancer [18], and bone loss related to aging [19] or cancer treatments [20, 21]. However, pharmacological therapies that clear or suppress senescent cells are in very early-stage clinical trials, with no “senotherapeutics” currently available for the treatment of osteoporosis or age-related diseases in humans.

Zoledronic acid, a commonly prescribed bisphosphonate for patients with osteoporosis or skeletal complications due to multiple myeloma or cancer metastases, may be a potential candidate drug for targeting cellular senescence. Zoledronic acid has an established safety profile and has been approved for clinical use for nearly 20 years [22]. Treatment with zoledronic acid reduces bone turnover, improves bone mineral density (BMD), and reduces fracture risk by as much as 70% [23]. Interestingly, zoledronic acid treatment has also been associated in individual studies with reduced risks of mortality [24, 25], cancer [26, 27], and cardiovascular disease [27–29]; however, a recent meta-analysis of 6 clinical trials of zoledronic acid failed to find a significant effect of zoledronic acid on overall mortality, although the authors acknowledged some uncertainty regarding these findings due to significant heterogeneity of the results [30]. Specifically, 2 large clinical trials of zoledronic acid treatment found 28% and 35% reductions in mortality [24, 26] that were not observed in other, smaller studies. Moreover, an earlier meta-analysis had demonstrated an overall reduction in all cause and cardiovascular mortality with use of bisphosphonates [29], although zoledronic acid was not specifically analyzed in that study.

Several mechanistic animal studies have shown that zoledronic acid treatment has beneficial non-skeletal effects, including increasing lifespan in a mouse model of Hutchinson-Gilford progeria syndrome [31], inhibition of tumor growth and metastasis [32, 33], cellular protection against radiation [34], and improved muscle function in mice after chemotherapy [35, 36]. Interestingly, these effects in models associated with DNA damage, a robust trigger for cellular senescence, are similar to those seen after senescent cell clearance. However, it is unclear if the skeletal or extra-skeletal effects of zoledronic acid are related to either senolytic (i.e. killing of senescent cells) or senomorphic (i.e. inhibition of the secretion of the SASP) effects or are unrelated to the senescence pathway. Thus, in the present study, we used multiple complementary approaches (*in vitro*, *in vivo*, and *in silico*) to evaluate possible effects of zoledronic acid on modulating cellular senescence.

## RESULTS

### Zoledronic acid has senolytic activity *in vitro*

**Figure 1:**
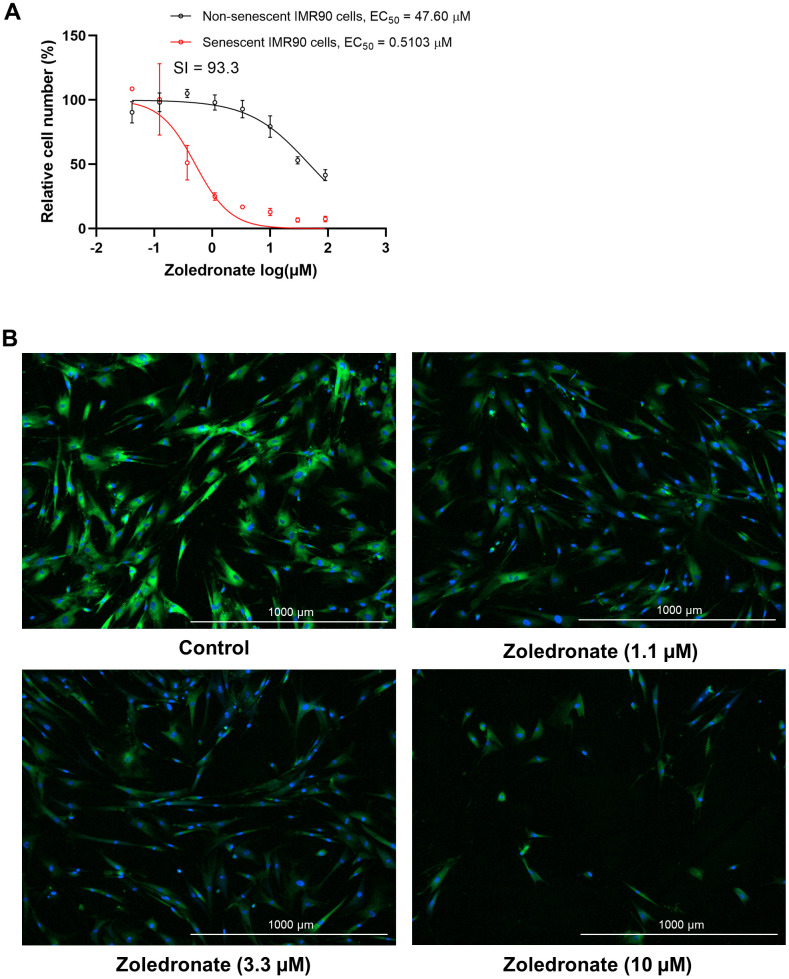
**Zoledronic acid has senolytic effects in human lung fibroblast IMR90 cells.** (**A**) Increasing concentrations (0.04-90 μM) of zoledronic acid were tested for 72 h in IMR90 cells. The figure shows the percentage non-senescent (black) and senescent IMR90 cells (red) remaining after 72 hours of treatment. n = 3; (**B**) Representative images of C_12_FDG-based senescence assay of zoledronic acid in senescent IMR90 cells. Blue fluorescence indicates nucleus staining with Hoechst 33324, and bright green fluorescence indicates SA-β-gal positive senescent cells whereas dim green fluorescence represents SA-β-gal low or negative, non-senescent cells. Images were taken using Cytation 1 at 4X.

To determine the effect of zoledronic acid on cellular senescence, human lung fibroblasts (IMR90 cells) that were induced to become senescent by etoposide were treated with increasing concentrations of zoledronic acid, and the percent of SA-β-gal+ senescent cells following treatment was determined by staining with the fluorogenic substrate 5-dodecanoylaminofluorescein di-β-D-galactopyranoside (C_12_FDG), as previously described [37, 38]. As shown in Figure 1A, zoledronic acid, in a dose range that is typically used to suppress osteoclast formation *in vitro* [39], exhibited minimal cytotoxicity towards non-senescent, proliferating IMR90 cells in comparison to senescent IMR90 cells, indicating that the senolytic effect of zoledronic acid was very specific to senescent cells. Moreover, the senolytic effect of zoledronic acid featured an extremely high selectivity index (SI) of 93.3, which is the ratio of half maximal effective killing concentration (EC_50_) values for non-senescent vs. senescent cells (Figure 1A). In general, an SI value ≥ 10 identifies a compound that is worthy of further investigation [40]. Figure 1B provides images of this selective elimination of senescent (SA-β-gal positive, green) cells by zoledronic acid in comparison to all cells (nucleus staining with Hoechst 33324, blue), These findings were extended to a second model system where DNA repair-deficient mouse embryonic fibroblasts (MEFs) from *Ercc1*-deficient (*Ercc1^-/-^*) mice [41] were generated. The *Ercc1^-/-^* MEFs were cultured at 20% O_2_ for 3 passages to induce senescence by oxidative stress where about half of the cells became senescent [37]. As shown in Supplementary Figure 1A, 1B, findings with the *Ercc1^-/-^* MEFs were very similar to those with the human lung fibroblasts. Taken together, these results demonstrate that zoledronic acid is a potent and selective senolytic in both human and mouse cells *in vitro*.

### Effects of zoledronic acid on circulating SASP factors and markers of frailty in aged mice

**Figure 2:**
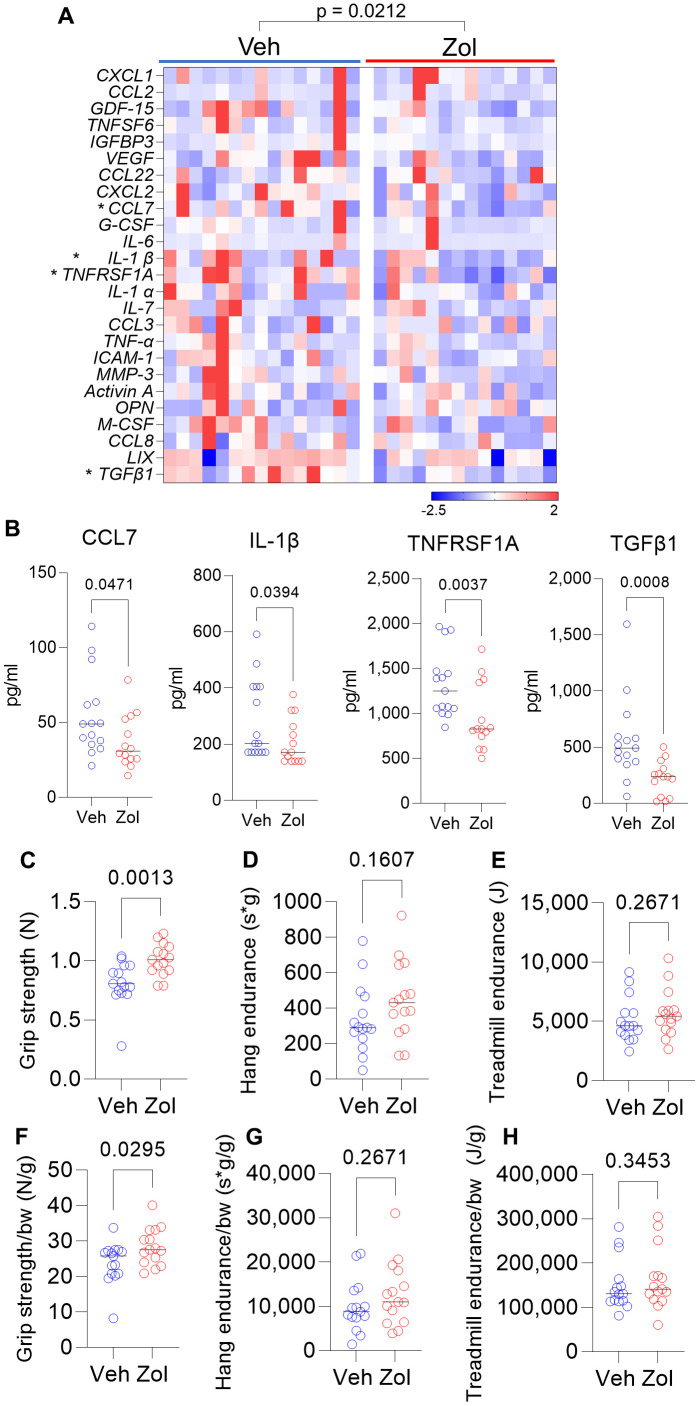
**Zoledronic acid reduces circulating SASP markers and improves grip strength in old mice.** (**A**) Zoledronic acid significantly reduces a panel of SASP markers in old mice (overall *p*-value is using the harmonic mean approach (see Methods)); (**B**) changes in serum CCL7, IL1β, TNFRSF1A, and TGFβ1 in the vehicle- vs. zoledronic acid- treated mice; effects of zoledronic acid on (**C**) grip strength; (**D**) hang endurance; and (**E**) treadmill endurance. (**F**–**H**) show these parameters normalized to body weight. *p*-values are using the Mann-Whitney test; n = 15 Veh, n=14 Zol mice/group (**A**, **B**) and n=15 per group (**C**–**H**).

Based on the above *in vitro* data, we next evaluated whether possible senolytic effects of zoledronic acid would be reflected by a reduction in circulating SASP proteins *in vivo* in mice. We found that in 24 month old mice who had been treated with zoledronic acid for 8 weeks, there was a significant overall reduction (harmonic mean *p*-value = 0.0212, see Statistical Methods) in a panel of established SASP proteins [7, 42, 43] in zoledronic acid- vs. vehicle-treated mice (Figure 2A), with individually significant reductions in CCL7, IL-1β, TNFRSF1A, and TGFβ1 (Figure 2B). The potential biological consequence of the reduction in circulating SASP factors was assessed by physical function testing, which revealed that grip strength improved significantly with zoledronic acid as compared to vehicle treatment (Figure 2C). However, we did not observe significant improvements in hang endurance or treadmill endurance in the zoledronic acid- vs. vehicle-treated mice (Figure 2D, 2E). Findings were similar when these parameters were normalized by body weight (Figure 2F–2H). In a separate cohort treated identically (see Methods), we obtained muscle weights, which did not differ between groups (Supplementary Figure 2A, 2B) as well as myofiber cross-sectional areas (CSA) at the quadriceps muscle (Supplementary Figure 2C–2E), which were also unchanged following zoledronic acid treatment. As shown in Supplementary Figure 3A–3C, there were no significant changes in body weight, lean mass, or fat mass in either the zoledronic acid- or vehicle-treated mice over the 8 weeks of treatment.

### Skeletal effects of zoledronic acid

As expected, spine trabecular (Supplementary Figure 4A–4D) and femur cortical (Supplementary Figure 4E) parameters were enhanced in the zoledronic acid- vs. vehicle-treated mice. Both serum CTx (marker of bone resorption, Supplementary Figure 4F) and P1NP (marker of bone formation, Supplementary Figure 4G) decreased in the zoledronic acid-treated mice but remained unchanged in the vehicle-treated mice. As previously described in rodents treated with zoledronic acid [44, 45], osteoclast numbers did not differ between groups (Supplementary Figure 4H); however, there was a marked reduction in osteoclasts that were attached to the bone surface (Supplementary Figure 4I) and a concomitant increase in detached osteoclasts (Supplementary Figure 4J, 4K), as has also been previously demonstrated in rodent models [44, 45]. Zoledronic acid treatment did not result in changes in osteoblast numbers (Supplementary Figure 4L) but did reduce mineral apposition (Supplementary Figure 4M) and bone formation (Supplementary Figure 4N) rates, reflecting the known coupling between bone resorption and bone formation [46]. Thus, treatment with zoledronic acid not only reduced circulating SASP factors and improved grip strength, but also had the expected effects on improving skeletal parameters in old mice.

**Figure 3:**
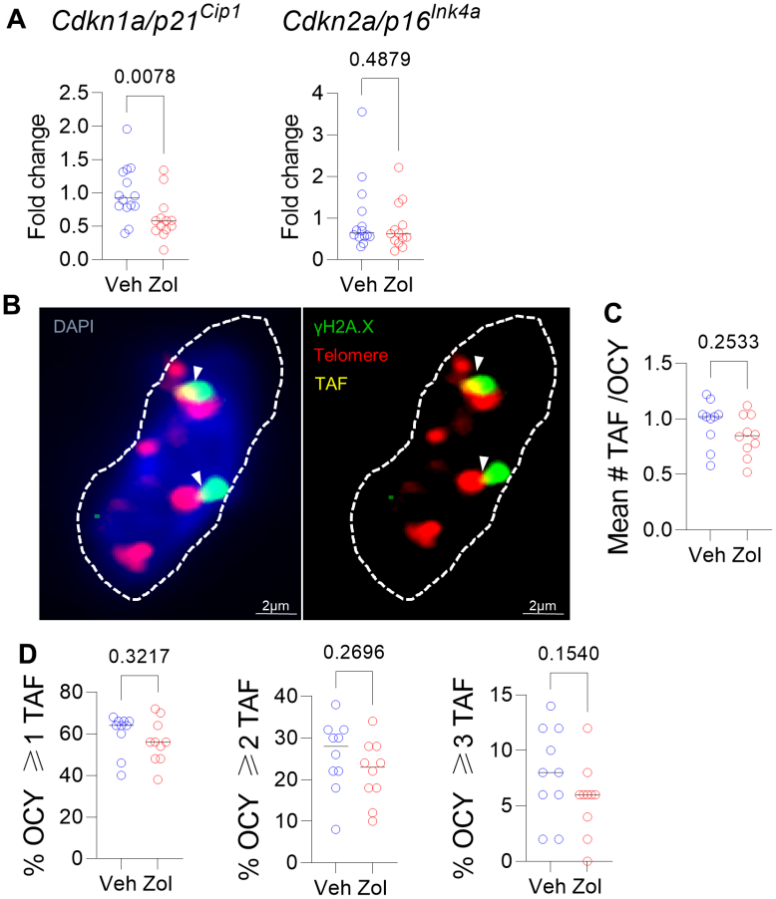
**Effects of zoledronic acid on senescence markers in bone.** (**A**) Zoledronic acid treatment led a significant downregulation of *Cdkn1a/p21^Cip1^*, but not *Cdkn2a/p16^Ink4a^* in the centrifuged metaphyses, enriched for osteocytes (see Methods). (**B**) Example of an osteocyte with γH2A.X (green) and telomeres (red); where they colocalize, a TAF is scored (yellow). (**C**) Mean #TAF/osteocyte, (**D**) percentage of osteocytes with ≥ 1, 2, or 3 TAF/osteocyte. *p*-values are using Mann-Whitney test; n = 14 Veh and n=13 Zol mice per group (**A**, **B**) and n=10/group (**B**–**D**).

Given the *in vitro* senolytic effects of zoledronic acid as well as the significant reduction in circulating SASP factors in the zoledronic acid-treated mice, we next examined the bones of vehicle- vs. zoledronic acid-treated mice for differences in senescence markers. As shown in Figure 3A, zoledronic acid treatment did result in a significant reduction in mRNA levels of one of the drivers of cellular senescence, *Cdkn1a/p21^Cip1^*, but not of *Cdkn2a/p16^Ink4a^,* in osteocyte-enriched bones from zoledronic acid- vs. vehicle-treated mice. Assessment of telomere-associated foci (TAF), a marker of telomeric DNA damage associated with senescence [47] (Figure 3B), showed an overall pattern of lower mean #TAF/osteocyte (Figure 3C) as well as the percentage of osteocytes with ≥ 1, 2, or 3 TAFs/cell (Figure 3D), but none of these differences were statistically significant. Statistically combining these parameters in a generalized mixed effects model resulted in a treatment *p*-value for zoledronic acid effects on TAFs of 0.161.

### Effects of zoledronic acid on bone marrow pre-osteoclastic cells

**Figure 4:**
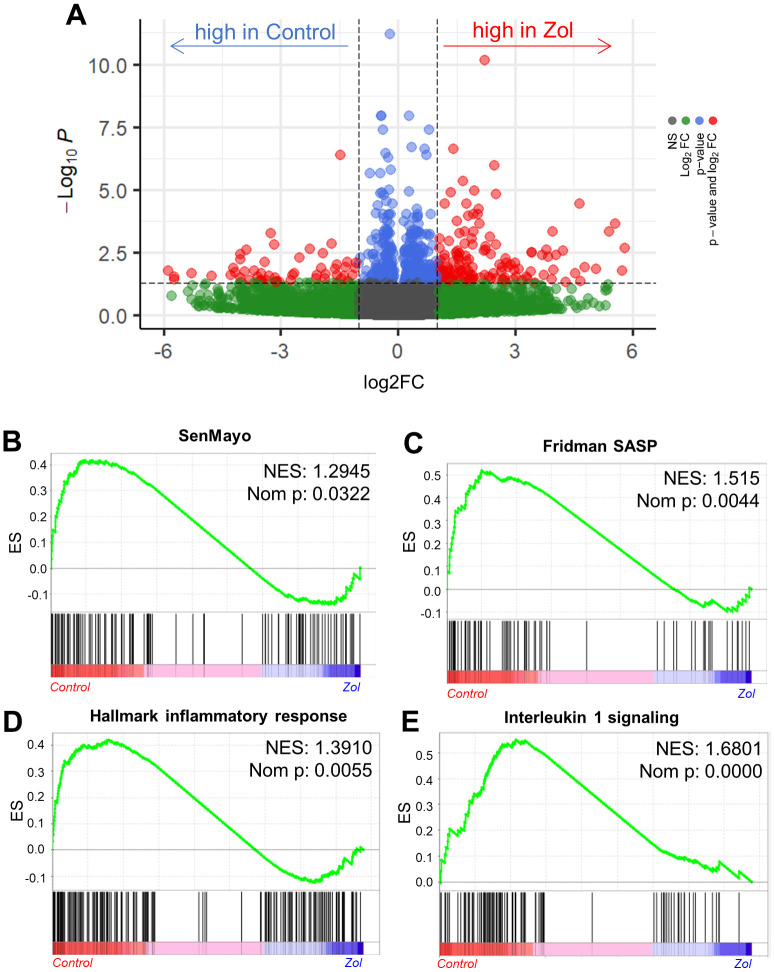
***In silico* analysis indicates the senolytic/senomorphic activity of zoledronic acid.** (**A**) Volcano plot showing the differentially regulated genes between control and zoledronic acid treatments. (**B**) The SenMayo and (**C**) Fridman senescene/SASP gene sets indicate a downregulation of senescence-associated pathways in the zoledronic acid group, and (**D**) the hallmark of inflammatory response as well as (**E**) Interleukin 1 signaling also indicate a lower inflammatory activity in the zoledronic acid group. *p*-values are using one-way ANOVA with multiple comparisons, adjusted with the Tukey *post-hoc* method. N=8 mice in the Control and n=7 in the zoledronic acid group.

As zoledronic acid executes its skeletal anti-resorptive actions by inhibiting bone resorption, we next investigated the potential senotherapeutic effects of zoledronic acid on osteoclast lineage cells. We first made use of a publicly available RNAseq dataset by Ubellacker et al. [48] (GSE108250) where C57BL/6J mice were treated for 5 days either with 50 μg/kg G-CSF, which is known to induce osteoclastogenesis *in vivo* [49, 50] (control), or a combination of 50 μg/kg G-CSF and 100μg/kg zoledronic acid. After isolation of pre-osteoclastic CD115+ (CSF1R/c-fms+) cells [51, 52] from the treated mice, RNA-sequencing of these cells was performed. Figure 4A shows a volcano plot of the differentially regulated genes in the control vs. zoledronic acid groups. To specifically evaluate effects of zoledronic acid on senescence/SASP gene expression, we used a recently validated gene set from our laboratory (SenMayo) that was demonstrated to identify senescent cells across tissues and species with high fidelity [53]. As shown in Figure 4B, this gene set was significantly downregulated in the CD115+ cells following zoledronic acid treatment, with similar findings noted using another publicly available SASP gene set (Fridman [54]; Figure 4C). Additional highly downregulated gene sets by zoledronic acid included the inflammatory response (Figure 4D) and IL1 signaling (Figure 4E) pathways. These *in silico* analyses thus demonstrate that zoledronic acid downregulates senescence/SASP and inflammatory pathways in pre-osteoclastic cells.

### Pre-osteoclastic cells are targeted by zoledronic acid treatment as demonstrated by cytometry by time of flight (CyTOF)

**Figure 5:**
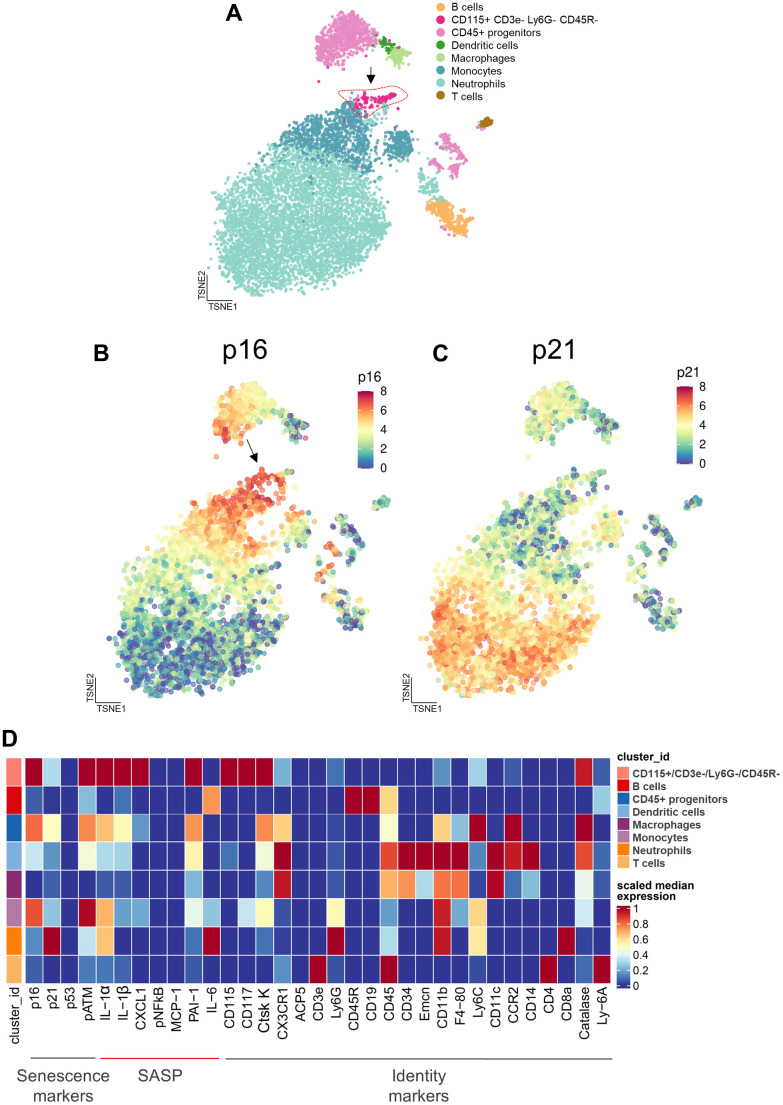
**Cytometry by time of flight (CyTOF) reveals the cellular targets of zoledronic acid treatment.** (**A**) CyTOF of hematopoietic cells following 2 weeks of zoledronic acid treatment led to distinct clusters. A t-distributed Stochastic Neighbor Embedding (tSNE) visualization of vehicle treated specimens (n=10) shows the integration of the datasets with the arrow pointing to the CD115+/CD3e-/Ly6G-/CD45R- cells. (**B**) p16 expression and (**C**) p21 expression in a tSNE visualization in the Veh group demonstrates a high expression of p16 in the CD115+ cells.vs. (**D**) Heatmap showing the expression of senescence, SASP, and identity markers in the bone marrow hematopoietic cells, demonstrating that the CD115+/CD3e-/Ly6G-/CD45R- cells express the highest levels of p16, pATM (DNA damage marker), and SASP proteins.

**Figure 6:**
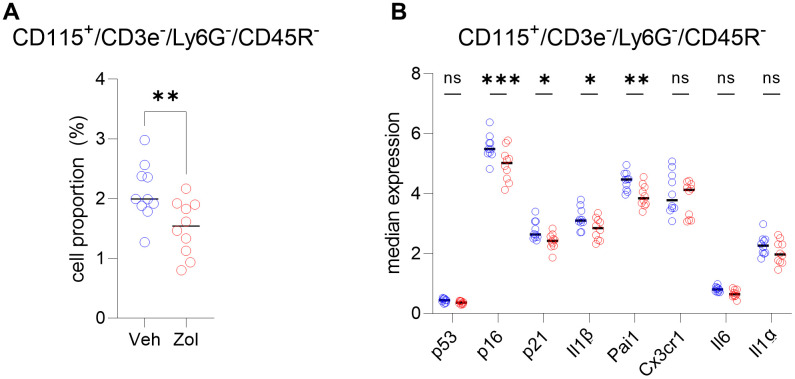
**CD115^+^ cells are targeted by zoledronic acid.** (**A**) Percent CD115^+^ cells are significantly reduced after zoledronic acid treatment (unpaired t-test, *p*<0.001). (**B**) The median expression in the vehicle vs. zoledronic acid samples shows a significant downregulation of p16 (*p*=0.0001), p21 (p<0.05) and the SASP markers IL1B (p<0.05) and PAI1/SERPINE1 (*p*<0.001) (two-way ANOVA with FDR correction for multiple comparisons, method by Benjamini, Krieger and Yekutieli). N=10 mice in the control and n=10 in the zoledronic acid group.

In order to extend the above RNAseq findings of Ubellacker et al. [48] we used a single cell proteomic approach (cytometry by time of flight, CyTOF). Supplementary Table 1 provides a list of all antibodies used in this analysis; note also that each of these antibodies have been validated for CyTOF by the Mayo Clinic CyTOF Core Laboratory, and additional validations of these antibodies in our laboratory are described in Doolittle et al. [55]. For these studies, we treated 21-month-old female mice with zoledronic acid or vehicle for two weeks. After isolation of the hematopoietic cell population, a total of 7,371,979 cells were analyzed by CyTOF. Figure 5A shows the expression profile of the identified clusters, and the FlowSOM clustering algorithm [56] revealed one population to be high in CD115 (Figure 5A, arrow). This cluster, which was negative for T cell (CD3e), neutrophil (Ly6G), and B cell (CD45R) markers, also had the highest expression of p16, CD117 (cKit), and Cathepsin K; p21 was present but not as high as in some other cell types (*e.g.,* neutrophils; Figure 5B–5D). Further, as shown in Figure 5D, these CD115+ cells also expressed high levels of the DNA damage marker, phospho-ATM [57], and were the most highly SASP-positive hematopoietic cells in the aged bone marrow microenvironment, expressing high levels of IL-1α, IL-1β, CXCL1, and PAI-1. Importantly, zoledronic acid clearly targeted these pre-osteoclastic cells, as this population (CD115+/CD3e-/Ly6G-/CD45R-) was significantly reduced following zoledronic acid treatment (by 29%, Figure 6A). This effect of zoledronic acid was specific for the CD115+ cells as zoledronic acid did not reduce the percentages of B- or T-cells, neutrophils, monocytes, or dendritic cells (Supplementary Figure 5A–5E). Moreover, the key senescence markers p16 as well as p21 were significantly downregulated in CD115+ cells, as were the SASP markers IL1-β and PAI1 (*Serpine1*) (Figure 6B). These findings thus demonstrate that the targets of zoledronic acid within the bone microenvironment are CD115+ cells which are reduced both in number and in their expression of key senescence/SASP markers following zoledronic acid treatment.

## DISCUSSION

In the present study, we used multiple, complementary approaches to evaluate the possible effects of zoledronic acid on cellular senescence. The evidence we provide that the beneficial extra-skeletal effects of zoledronic acid may be mediated, at least in part, by modulation of cellular senescence is the following: (1) *In vitro*, zoledronic acid exhibited potent senolytic effects with a high selectivity index on both human and mouse senescent cells; (2) *in vivo,* in aged mice, treatment with zoledronic acid was associated with a significant reduction in a panel of circulating SASP factors concomitant with an improvement in grip strength, although we acknowledge that other markers of frailty were not improved by zoledronic acid; (3) we complemented these findings with *in silico* analysis of an independent study and model by Ubellacker et al. [48] that included RNAseq analysis of pre-osteoclastic CD115+ (CSF1R/c-fms+) cells [51, 52], which revealed a significant reduction in two panels of senescence/SASP genes (SenMayo and Fridman) in these cells by zoledronic acid; and (4) we directly tested for possible senolytic/senomorphic effects of zoledronic acid using single cell proteomic analysis (CyTOF) and demonstrated that within the hematopoietic bone microenvironment, a subset of pre-osteoclastic cells (CD115+/CD3e-/Ly6G-/CD45R-) expressed high levels of p16 and SASP markers and that these cells were specifically reduced by zoledronic acid, along with a decrease in the expression of several SASP markers. However, the reduction of the pre-osteoclastic population did not directly result in a reduction in total osteoclast numbers, potentially due to a heterogeneous precursor population (CD115+/CD3e-/Ly6G-/CD45R-) that includes not just osteoclast precursors but also other cell types. Nonetheless, the number of detached osteoclasts was significantly increased and the number of attached osteoclasts was significantly decreased, consistent with a functional effect of zoledronic acid on mature osteoclasts.

Our finding that myeloid cells expressing CSF1R (CD115+) are specifically targeted by zoledronic acid and are the most highly enriched hematopoietic cells in senescence/SASP/inflammatory genes and proteins are of considerable interest in light of the demonstration by Ambrosi and colleagues [58] that the ligand for CSF1R, *Csf1*, was highly upregulated in skeletal stem cells from aged (24 month) compared with young (2 month) mice. Thus, the targeting of the CSF1R+ cells by zoledronic acid may be particularly important in terms of interrupting an inflammatory loop between aging skeletal stem cells and myeloid cells, thereby contributing to the beneficial non-skeletal effects of zoledronic acid. We should note that the reduction in senescence/SASP markers in the CD115+/CD3e-/Ly6G-/CD45R- cells is not due simply to a reduction in cell numbers, as the senescence/SASP markers were normalized to cell numbers (see Methods). In addition, whether the reduction in SASP markers leads to the reduction in the CD115+ cell population is something we cannot address based on our current data; this would require additional *in vitro* mechanistic studies.

It is of interest that, consistent with our findings showing senescent pre-osteoclasts as key targets of zoledronic acid, work by Su et al. [59] has identified an apparently very similar population of pre-osteoclastic cells expressing senescence/SASP markers that accumulate in subchondral bone in a mouse model of osteoarthritis in the setting of a high fat diet. In that study, inhibiting the SASP from these cells attenuated the progression of the osteoarthritis. Moreover, previous work from the same laboratory also demonstrated that aged mice fed a high fat diet had high serum PDGF-BB levels that were derived, in fact, from bone marrow pre-osteoclastic cells [60] and these investigators linked the increased PDGF-BB to bone loss as well as arterial stiffening. Thus, there is an evolving body of evidence implicating pre-osteoclastic cells expressing senescence/SASP markers as playing important roles in mediating tissue dysfunction in various settings. We should note, however, that senescence was originally described in the context of mesenchymal cell populations [61], and further studies are needed to compare features of senescence in mesenchymal cells vs. the apparent senescent phenotype of immune/hematopoietic cell populations.

In terms of mesenchymal cells, we did demonstrate potent senolytic effects of zoledronic acid on senescent human lung and mouse embryonic fibroblast cells *in vitro*. *In vivo*, zoledronic acid treatment reduced *Cdkn1a/p21^Cip1^,* but not *Cdkn2a/p16^Ink4a^*, mRNA levels in osteocyte-enriched bone fractions from zoledronic acid- compared to vehicle-treated mice. Using the highly specific TAF assay for telomeric DNA damage, we did observe a trend for reduced TAFs in osteocytes with zoledronic acid treatment, but the differences did not reach statistical significance. Thus, although our findings clearly implicate myeloid CD115+ cells as bona fide targets of the senotherapeutic and/or anti-inflammatory effects of zoledronic acid *in vivo*, it is possible that zoledronic acid also targets senescent mesenchymal cells, and further studies are needed to define the senescent cell populations *in vivo* more clearly that may be reduced by zoledronic acid.

Our findings in mice are also consistent with recent findings in *Drosophila* demonstrating that zoledronic acid treatment was associated with an extension of lifespan and improved climbing ability, which is analogous to assessment of grip strength in mice [62]. This study also linked the known inhibition of farnesyl pyrophosphate synthetase (FPPS) by zoledronic acid to a reduction in the accumulation of DNA damage, which is a key trigger of cellular senescence [10]. These findings in *Drosophila* are similar to previous work from the same group demonstrating that zoledronic acid extended the lifespan of human bone marrow stromal cells (BMSCs) *in vitro*, and this was associated with a reduction in markers of DNA damage as well as reduced p21 and p16 protein expression [34]. Whether, similar to the findings in the *Drosophila* model [62], the inhibition of FPPS by zoledronic acid also mediates the reduction of the CD115+ cells and the senescence/SASP markers in this study remains unclear and warrants further study.

Although our studies, when combined with the previous work noted above, point to potential effects of zoledronic acid on modulating cellular senescence and the SASP, we recognize important limitations. Specifically, although in this “proof-of-concept” study we administered zoledronic acid twice weekly to mice using a well-established regimen for rodent studies [63], patients with osteoporosis are generally only treated with zoledronic acid once annually. However, it is possible that ongoing release of zoledronic acid from the skeleton, as has been demonstrated to occur [64], may not only inhibit osteoclastic bone resorption but also affect the highly inflammatory CD115+ cells we describe here. Because these cells are present in the bone microenvironment, zoledronic acid may reduce both their number and secretion of SASP/inflammatory factors, thereby leading to beneficial physiological effects. Under this scenario, it is possible that the effects of zoledronic acid on reducing the SASP of the CD115+ cells may contribute to its beneficial systemic effects. However, given the strong targeting of zoledronic acid to the skeleton [64], analogs of zoledronic acid with a broader tissue distribution than zoledronic acid that yet retain its senolytic effects may well be more useful in terms of ameliorating not just bone loss, but other age-related co-morbidities. Moreover, given the issues related to cancer- and chemotherapy-related frailty, potential benefits of zoledronic acid may be most relevant, at least initially, to cancer patients.

We also note that due to cost and availability of aged mice, we only studied female mice; while this is consistent with the epidemiology of osteoporosis in that fractures in women far outnumber those in men [65] and thus far more women than men are treated with zoledronic acid, additional studies are needed to evaluate possible sex differences in the effects of zoledronic acid on senescence and SASP markers we observed. In addition, we studied the mice following 8 weeks of treatment with vehicle or zoledronic acid and found a significant improvement in grip strength; it is possible that a longer duration of treatment (or a longer time interval following the last dose) may have led to increases in other measures of frailty (*e.g.,* treadmill endurance, others). These limitations notwithstanding, given our *in vitro* data of senolytic effects of zoledronic acid and corroboratory *in vivo* findings, our work should provide an impetus to develop zoledronic acid (or other bisphosphonate) analogs with greater senolytic efficacy as well as distribution to non-skeletal tissues for beneficial effects not only in bone, but across multiple aging organs.

## MATERIALS AND METHODS

### Cell culture

Human lung fibroblast IMR90 cells were obtained from American Type Culture Collection (ATCC) and cultured in EMEM medium supplemented with 10% fetal bovine serum. To induce senescence, IMR90 cells were treated with 20 μM etoposide for 24 h. After etoposide removal, cells were cultured in normal medium for additional 5 days before being collected for senescence assay. Primary *Ercc1^-/-^* mouse embryonic fibroblasts (MEFs) were isolated on embryonic day 12.5-13.5. In brief, mouse embryos were isolated from yolk sac followed by the complete removal of viscera, lung, and heart if present. Embryos were then minced into fine chunks, fed with MEFs medium, and cultivated under 3% O_2_ to reduce stresses. Cells were split at 1:3 when reaching confluence. MEFs were grown at a 1:1 ratio of Dulbecco’s Modification of Eagles Medium (supplemented with 4.5 g/L glucose and L-glutamine) and Ham’s F10 medium, supplemented with 10% fetal bovine serum, penicillin, streptomycin, and non-essential amino acid. To induce oxidative stress-mediated DNA damage, *Ercc1^-/-^* MEFs were switched to 20% O_2_ for 3 passages [37].

### SA-β-gal senescence assay by C_12_FDG staining

Senescent IMR90 cells or *Ercc1^−/−^* MEFs were seeded at 2000 cells per well in black walled, clear bottomed 96-well plates at least 6 h prior to treatment. Following the addition of zoledronic acid or control, the IMR90 cells were incubated for 72 h and the *Ercc1^−/−^* MEFs for 48 h at 20% O_2_. After removing the medium, cells were incubated in 100 nM Bafilomycin A1 in culture medium for 1 h to induce lysosomal alkalinization. Then 10 μM or 20 μM of fluorogenic substrate C_12_FDG (Setareh Biotech, Eugene, OR, USA) were added to IMR90 cells or MEFs, respectively, and incubated for 2 h, followed by counterstaining with 2 μg/ml Hoechst 33342 (Thermo Fisher Scientific, Waltham, MA, USA) for 15 min. Subsequently cells were washed with PBS and fixed in 2% paraformaldehyde for 15 min. Finally, cells were imaged with 6 fields/well using a high content fluorescent image acquisition and analysis platform Cytation 1 (BioTek, Winooski, VT, USA) [37, 38].

### Study approval

Animal studies were performed under protocols approved by the Institutional Animal Care and Use Committee (IACUC) and experiments were performed in accordance with Mayo Clinic IACUC guidelines.

### Animal studies

The study included three mouse cohorts. Note that in order to maximize power and given that zoledronic acid is used in a far greater proportion of women than men, we only studied female mice. The first cohort included 22-month-old female *C57BL/6N* mice (n=15/group) that were treated intraperitoneally with zoledronic acid (125 μg/kg) or vehicle twice weekly for 8 weeks, using a regimen established by Dr. Russell and the Sheffield group [63]. In this cohort we assessed body composition, circulatory SASP factors, bone turnover markers, frailty, skeletal μCT analysis, qPCR analysis of metaphyseal bone samples, and TAFs. The second cohort was to assess muscle weights and muscle fiber cross-sectional area. In this cohort, 21-month-old female *C57BL/6N* mice (n=10/group) were treated intraperitoneally with the same zoledronic acid regimen (125 μg/kg twice weekly) or vehicle for 8 weeks. Finally, for the cytometry by time of flight (CyTOF) studies, mice were treated for two weeks with either zoledronic acid (n=10) or vehicle (n=10), the mice were sacrificed, and one mouse tibia isolated. All mice were housed in ventilated cages in a pathogen-free, accredited facility under a 12-hour light/12-hour dark cycle with constant temperature (23° C) and access to food and water *ad libitum*. All assessments were performed in a blinded fashion.

### Assessment of body composition

Body mass (grams) was recorded on all mice at the onset of the study and 2, 4, and 6 weeks after zoledronic acid or vehicle treatment and finally at the endpoint (week 8). Body composition (i.e. whole-body lean grams and fat mass grams) was assessed at the baseline and endpoint of the study *in vivo* in non-anesthetized and conscious mice using quantitative Echo magnetic resonance imaging (EchoMRI-100), as described [66].

### Mouse tissue collections

Prior to sacrifice, body mass was recorded in the morning (under nonfasting conditions) and serum/plasma was collected from anesthetized mice by cardiac puncture at time of death and stored at –80° C. After euthanasia, the tibiae, femurs, humeri, and vertebrae were excised from the mice and skeletal muscle/connective tissues were removed. The right femur and part of the lumbar vertebrae (L4–6) were fixed in ethanol (EtOH) for *ex vivo* μCT scanning followed by histomorphometry. Another part of the lumbar vertebrae (L1-3) were embedded in methyl methacrylate and sectioned for TAFs. The metaphysis of the R tibia was cut and centrifuged to remove bone marrow and obtain an osteocyte-enriched sample for further analysis, as previously described [67]. The samples were immediately homogenized in QIAzol Lysis Reagent (Qiagen) and frozen at –80° C for rt-qPCR mRNA gene expression analyses.

### Measurement of circulating SASP factors

The concentrations of CCL2, CCL3, CCL7, CCL8, CCL22, CXCL1, CXCL2, G-CSF, GDF-15, ICAM-1, IGFBP3, IL-1α, IL-1β, IL-7, IL-17, LIX, M-CSF, MMP-3, OPN, TNFSF6, TNFRSF1A, TNF-α, and VEGF in plasma were quantified using commercially available multiplex magnetic bead immunoassays (R&D Systems) based on the Luminex xMAP multianalyte profiling platform and analyzed on a MAGPIX System (Merck Millipore). All assays were performed according to the manufacturer’s protocols. Activin A and TGF-β1 concentration were determined by a Quantikine ELISA Kit (R&D Systems) according to the manufacturer’s instructions. Investigators were blinded as to sample identity and treatment of mice.

### Assessment of physical function

All measurements were performed at least 5 days after the last dose of zoledronic acid or vehicle treatment as previously described [13]. Forelimb grip strength (N) was determined using a Grip Strength Meter (Columbus Instruments, Columbus, OH, USA). Results were averaged over ten trials. For the hanging test, mice were placed onto a 2-mm-thick metal wire that was 35 cm above a padded surface. Mice were allowed to grab the wire with their forelimbs only. Hanging time was normalized to body weight as hanging duration (sec) × body weight (g). Results were averaged from 3 trials for each mouse. For treadmill performance, mice were acclimated to a motorized treadmill at an incline of 5° (Columbus Instruments) over 3 days for 5 min each day, starting at a speed of 5 m/min for 2 min and progressing to 7 m/min for 2 min and then 9 m/min for 1 min. On the test day, mice ran on the treadmill at an initial speed of 5 m/min for 2 min, and then the speed was increased by 2 m/min every 2 min until the mice were exhausted. Exhaustion was defined as the inability to return onto the treadmill despite a mild electrical shock stimulus and mechanical prodding. Distance was recorded and total work (kJ) was calculated using the following formula: mass (kg) × g (9.8 m/s^2^) × distance (m) × sin (5°).

### Evaluation of myofiber cross-sectional areas

Quadriceps muscles were embedded in Optimal Cutting Temperature compound (Sakura Finetek, Torrance, CA, USA), frozen in liquid nitrogen-cooled 2-methylbutane (Sigma, St. Louis, MO, USA), and stored at -80° C. Transverse 7 μm-thick frozen sections were cut with a Leica cryostat and mounted onto SuperFrost Plus slides (Thermo Fisher Scientific, Waltham, MA, USA). Muscle sections were dried for 2 hours at -20° C, and then stored at -80° C until analysis. Frozen quadriceps sections were removed from -80° C and fixed in 4% paraformaldehyde (PFA) for 15 minutes. After being permeabilized and blocked, the sections were incubated with rabbit anti-laminin antibody (L9393, Sigma-Aldrich) overnight at 4° C, followed by goat-anti-rabbit cross-adsorbed secondary antibody Alexa Fluor 488 (A11008, Invitrogen by Thermo Fisher Scientific) for 1 hour in a humidified chamber at room temperature. Sections were mounted using ProLong Gold Antifade Mountant with DAPI (Invitrogen by Thermo Fisher Scientific). Images were captured at 10x magnification with a Zeiss Axio Imager microscope. Myofiber cross-sectional area, demarcated by Laminin staining, was quantified using MuscleJ [68].

### Skeletal μCT analysis

All imaging and analysis were performed in a blinded fashion as described by our group previously [19, 42]. At study endpoint, *ex vivo* quantitative analyses of bone microarchitecture of the lumbar vertebrae (L5) and right femur (mid-shaft diaphysis) were performed. Scan settings were as follows: 55 kVp, 10.5 μm voxel size, 21.5 diameter, 145mA, 300 ms integration time. μCT parameters were derived using the manufacturer’s protocols. Trabecular bone parameters were at the lumbar spine (200 slices). Furthermore, at the mid-diaphysis (50 slices) of the right femur, cortical thickness (Ct.Th; mm) was assessed.

### Bone turnover marker measurements

All biochemical assays for bone turnover markers (P1NP and CTX) were performed in blinded fashion. At the study endpoint, serum was collected in the morning (under non-fasting conditions) from anesthetized mice by cardiac puncture and stored at -80° C in aliquots for additional biochemical assays, which included circulating levels of bone turnover markers. Specifically, the serum bone formation marker P1NP (amino-terminal propeptide of type I collagen; ng/mL) was measured using the rat/mouse P1NP enzyme immunoassay (EIA) kit (interassay coefficient of variation [CV] <10%), and the serum bone resorption marker CTx (cross-linked C-telopeptide of type I collagen; ng/mL) was measured using a RatLaps Rat/Mouse CTx EIA kit (interassay CV <10%). Kits were purchased from Immuno Diagnostic Systems (IDS, Scottsdale, AZ, USA).

### Bone histomorphometry

All histomorphometric analyses were performed in a blinded manner. For dynamic histomorphometry, mice were injected subcutaneously with Alizarin Red (0.1mL/animal, 7.5mg/mL) and calcein (0.1 mL/animal, 2.5mg/mL) on days 9 and 2, respectively, before euthanasia. The lumbar vertebrae were isolated from WT mice treated with vehicle or zoledronic acid. The vertebrae were embedded in MMA, sectioned, and stained with Masson Trichrome to assess osteoblast numbers/bone perimeter (N.Ob/B.Pm,/mm), or stained for tartrate-resistant acid phosphatase (TRAP) activity to assess osteoclast numbers per bone perimeter, N.Oc/B.Pm,/mm). Alternatively, sections were left unstained to quantify trabecular mineralizing surfaces (mineral apposition rate, MAR, μ/d; bone formation rate/bone surface, BFR/BS, μm^3^/μm^2^/d), which were 100μm apart from the growth plate and 100 μm apart from the cortical anterior or posterior vertebral body perimeter. Osteoblast (N.Ob/B.Pm) and osteoclast (N.Oc/B.Pm) numbers were assessed at the same distance from cortical bone to verify trabecular assessments. All histomorphometric measurements and calculations were performed with the Osteomeasure Analysis system (Osteometrics, Atlanta, GA, USA).

### rt-qPCR analysis of metaphyseal bone

Osteocyte-enriched cell preparations were generated as described in Mouse tissue collections, immediately homogenized in QIAzol Lysis Reagent (Qiagen, Valencia, CA, USA), and stored at -80° C for subsequent RNA extraction, cDNA synthesis, and targeted gene expression measurements of mRNA levels by rt-qPCR, as described [69]. Total RNA was extracted according to the manufacturer’s instructions using QIAzol Lysis Reagent followed by purification with RNeasy Mini Columns (Qiagen). On-column RNase-free DNase solution (Qiagen) was then applied to degrade potentially contaminating genomic DNA. RNA purity/quantity was confirmed by Nanodrop spectrophotometry (Thermo Fisher Scientific, Wilmington, DE). Standard reverse transcriptase was performed using the High-Capacity cDNA Reverse Transcription Kit (Applied Biosystems by Life Technologies, Foster City, CA, USA). Transcript mRNA levels were determined by rt-qPCR on the ABI Prism 7900HT Real Time System (Applied Biosystems, Carlsbad, CA) using murine TaqMan assays (Thermo Fisher Scientific, Wilmington, DE, USA) to measure *p16^Ink4a^* (*Cdkn2a*) and *p21^Cip1^* (*Cdkn1a*), as described [69].

### Assessment of TAFs

To measure cellular senescence in vertebral trabecular bone, the TAF assay (*n* = 10/group) was performed on murine vertebral trabecular bone nondecalcified methyl methacrylate–embedded sections. Our protocol was adapted from a previous study [47]. Bone sections were deplasticized and hydrated in EtOH gradient followed by water and PBS. Antigen was retrieved by incubation in Tris-EDTA (pH 9.0) at 95° C for 15 minutes. After cooldown and hydration with water and PBS (0.5% Tween-20/0.1% Triton X-100), slides were placed in blocking buffer (1:60 normal goat serum; Vector Laboratories; S-1000; in 0.1% BSA/PBS) for 30 minutes at room temperature (RT). Primary antibody γ-H2AX (1:200; anti–γ-H2A.X rabbit monoclonal antibody, Cell Signaling Technology; 9718) was diluted in blocking buffer and incubated overnight at 4° C. The next day, slides were washed with PBS (0.5% Tween-20/0.1% Triton X-100) followed by PBS alone and then incubated for 30 minutes with secondary goat, anti-rabbit antibody biotinylated (1:200; Vector Laboratories; BA-1000) in blocking buffer. Subsequently, slides were washed with PBS (0.5% Tween-20/0.1% Triton X-100) followed by PBS alone, then incubated for 60 minutes with tertiary antibody (1:500; Cy5 Streptavidin, Vector Laboratories; SA-1500) in PBS. Slides were then washed 3 times with PBS, followed by FISH for TAF detection. Briefly, following 4% paraformaldehyde cross-linking for 20 minutes, sections were washed 3 times (5 minutes each in PBS) and dehydrated in graded (70%, 90%, and 100%, 3 minutes each) ice-cold EtOH. Sections were then dried and denatured for 10 minutes at 80° C in hybridization buffer: 0.1 M Tris (pH 7.2), 25 mM MgCl2, 70% formamide (MilliporeSigma), 5% blocking reagent (Roche), with 1.0 μg/mL of Cy3-labeled telomere-specific (CCCTAA) peptide nucleic acid probe (TelC-Cy3, Panagene Inc.; F1002), followed by humidified dark room hybridization for 2 hours at RT. Sections were then washed and mounted with VECTASHIELD DAPI-containing mounting medium (Life Technologies) before image acquisition and analysis. The number of TAF/osteocyte was quantified in a blinded fashion by examining overlap of signals from the telomere probe with γ-H2AX (i.e. phosphorylation of the C-terminal end of histone H2A.X — a marker of double-strand breaks in DNA). The mean number of TAF/osteocyte in vertebral trabecular bone was quantified using FIJI (an ImageJ distribution software; NIH, https://imagej.nih.gov/ij/), and the percentage of TAF^+^ OCYs was calculated for each mouse based on the following criteria: percentage of OCYs with ≥1 TAF, percentage of OCYs with ≥2 TAF, and percentage of OCYs with ≥3 TAF.

### *In silico* analysis of zoledronic acid effects on pre-osteoclastic cells

For this analysis, we used publically available data from Ubellacker et al. [48] (GSE108250). Briefly, in those studies, transcriptome-wide gene expression data were generated from CD115^+^ (c-fms+) bone marrow cells (from femora and tibiae) from six to seven-week-old female C57BL/6J mice, treated with a single dose of 100μg/kg (i.p.) zoledronic acid (Novartis Pharmaceuticals) and/or 50 μg/kg (i.p.) recombinant human granulocyte-colony stimulating factor (G-CSF; carrier-free, BioLegend #578604) for three days. Sequencing was performed on a HiSeq2500 (Illumina^®^), fastq files were mapped to the murine reference genome mm10, and analysis was performed as previously described [70]. Significantly differentially regulated genes were selected by a Benjamini–Hochberg adjusted p-value ≤ 0.05 and log_2_-fold changes above 0.1 or below -0.1. Gene Set Enrichment Analysis (GSEA) [71, 72] was performed with default settings (1000 permutations for gene sets, Signal2Noise metric for ranking genes). Comparisons were made between OC precursors in combination therapy (zoledronic acid +G-CSF, n=7 and G-CSF alone, n=8). The analyses we present are new, and we focused on pathways and differentially expressed genes different from the ones in Uebellacker et al. [48] (GSE108250).

### Cytometry by time of flight (CyTOF)

After a two-week treatment with either zoledronic acid (n=10) or vehicle (n=10), the mice were sacrificed and one mouse tibia isolated. The epiphyseal region was removed, and the bone centrifuged. The resuspended bone marrow was RBC lysed, resuspended, and kept on ice. For labeling, the cells were incubated at 4° C with metal-conjugated antibodies (see. Supplementary Table 1). The cells were incubated with cell surface targeting antibodies for 45 min. Subsequently, they were fixed with 2% paraformaldehyde, washed, and incubated with antibodies for intracellular antigens for 45 min. After labeling, the cells were assayed on a Helios II mass cytometer (Fluidigm, South San Francisco, CA, USA).

The generated Fcs files were normalized and debarcoded with Cytobank (Beckman Coulter Life Sciences, Indianapolis, IN, USA). A manual selection of alive singlet cells was performed, and the resulting fcs files uploaded in R version 4.0.2 (The R Project for Statistical Computing). The subsequent analyses have been done with the packages HDCytoData (1.10.0), flowWorkspace (4.2.0), openCyto (2.2.0), CATALYST (1.14.1) and SingleCellExperiment (1.12.0), following the vignette provided by Nowicka et al. [73]. The expression of the senescence/SASP markers was normalized and scaled for total cell numbers in each population. The clustering followed the FlowSOM recommendations with a maximum of 20 clusters according to the “type” markers (see Supplementary Table 1).

Metal-conjugated antibodies used in this study are summarized in the Supplementary Table 1. Except for commercially available pre-conjugated antibodies (Fluidigm Sciences, Sunnyvale, CA, USA), all antibodies were conjugated to isotopically enriched lanthanide metals using the MaxPAR antibody conjugation kit (Fluidigm Sciences), according to the manufacturer’s recommended protocol. Labeled antibodies were stored at 4° C in PBS supplemented with glycerol, 0.05% BSA, and 0.05% sodium azide. All antibodies were tested with control beads as well as positive and negative control cells. Note that each of these antibodies have been validated for CyTOF by the Mayo Clinic CyTOF Core Laboratory; additional validations for the antibodies used are provided in Doolittle et al. [55].

### Statistical analyses

Graphical data are shown as medians with raw data points unless otherwise specified. The sample sizes were determined based on previously conducted and published experiments [19] in which statistically significant differences were observed among various bone parameters in response to multiple interventions in our laboratory. The animal numbers used are indicated in the figure legends; all samples presented represent biological replicates. The only samples excluded from analysis were the SASP factors from one mouse in the zoledronic acid group who uniformly had extremely high levels across multiple SASP factors, with many > 3 SDs beyond the mean of the other mice, indicating a systemic biological (i.e., unrecognized illness) or technical issue with the samples from that mouse. In agreement with our statistician (E.A.), this mouse was thus excluded from the SASP assays. The distribution of the data was examined using dot plots and histograms. Group comparisons were made using the non-parametric Mann-Whitney U test. The harmonic mean test [74] was used to combine the p-values from individual markers. The statistical analyses were performed using either GraphPad Prism (GraphPad Software, Inc., Version 9.0), R version 4.0.2 (The R Project for Statistical Computing), SPSS (IBM, Version 25) and GSEA (Broad Institute, V 4.1.0). Used R packages were EnhancedVolcano (1.10.0), gprofiler2 (0.2.0), clusterProfiler (version 3.10.1), and enrichplot (1.11.3). A *p*-value <0.05 (two-tailed) was considered statistically significant.

### Data availability

All source data will be provided upon request.

### Code availability

All relevant code information will be provided upon request.

## Supplementary Material

Supplementary Figures

Supplementary Table 1
