# Supplementary material for: In vitro and in vivo effects of zoledronic acid on senescence and senescence-associated secretory phenotype markers
Source: Aging (Albany NY). 2023 May 7;15(9):3331–55. doi: 10.18632/aging.204701 (PMC10449299; doi:10.18632/aging.204701)
Supplement: Supplementary Figures [file aging-15-204701-s001.pdf]

SUPPLEMENTARY FIGURES

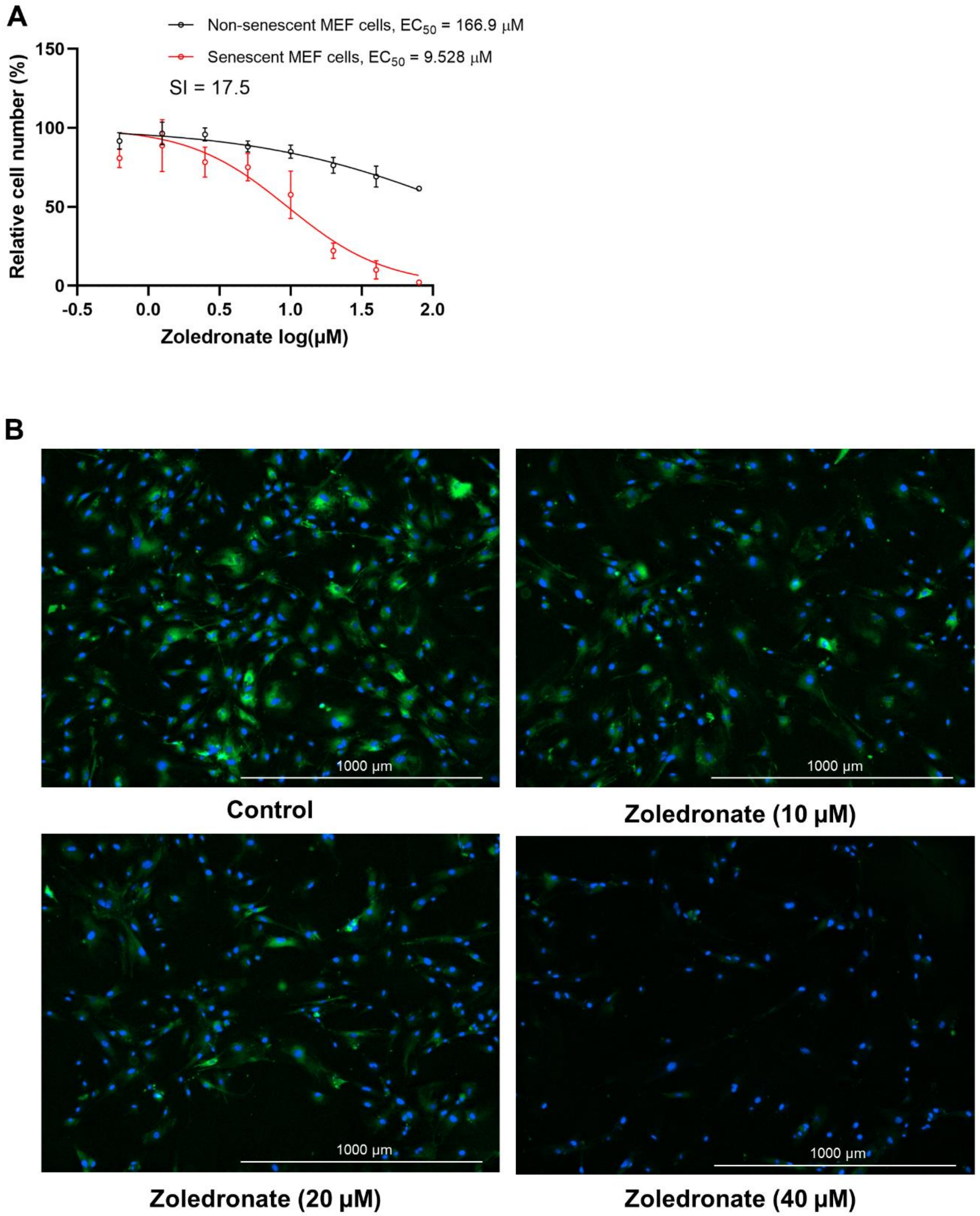

Supplementary Figure 1. Zoledronate has senolytic effects in mouse embryonic fibroblast (MEF) cells. (**A**) Increasing concentrations (0.63-80 μM) of zoledronate were tested for 48 h in MEF cells. The figure shows the percentage of WT, non-senescent MEF cells (black) and senescent *Ercc1*$^{-1-}$ MEF cells (red) remaining after 48 hours of treatment. SI: selectivity index. n = 3; (**B**) Representative images of C_{12}FDG-based senescence assay of zoledronate in senescent *Ercc1*$^{-/-}$ MEFs. Blue fluorescence indicates nucleus staining with Hoechst 33324, and bright green fluorescence indicates SA-β-gal positive senescent cells whereas dim green fluorescence represents SA-β-gal low or negative, non-senescent cells. Images were taken using Cytation 1 at 4X.

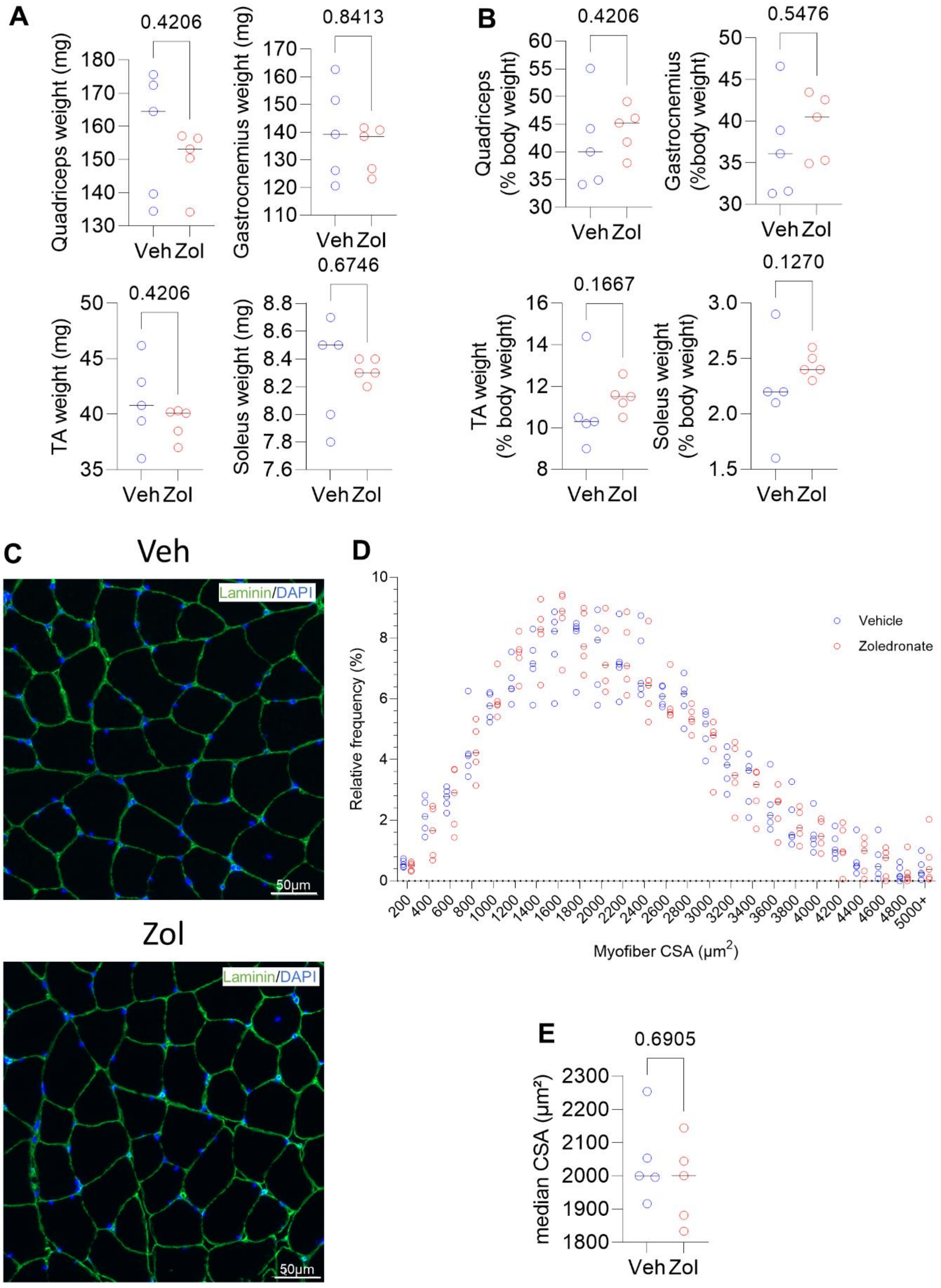

Supplementary Figure 2. Effects of zoledronate on muscle weight and muscle fiber cross-sectional area. (**A**) Quadriceps femoris, gastrocnemius, tibialis anterior (TA) and soleus muscle weights in the vehicle- and zoledronate-treated mice; (**B**) Muscle weights normalized to body weights; (**C**) Cross-section of quadriceps muscle fibers showing laminin and nuclear staining; (**D**) Myofiber CSA distribution in the vehicle- and zoledronate-treated mice; (**E**) Quadriceps muscle CSA in the vehicle- and zoledronate-treated mice. *p*-values according to Mann-Whitney test, n=5 mice per group.

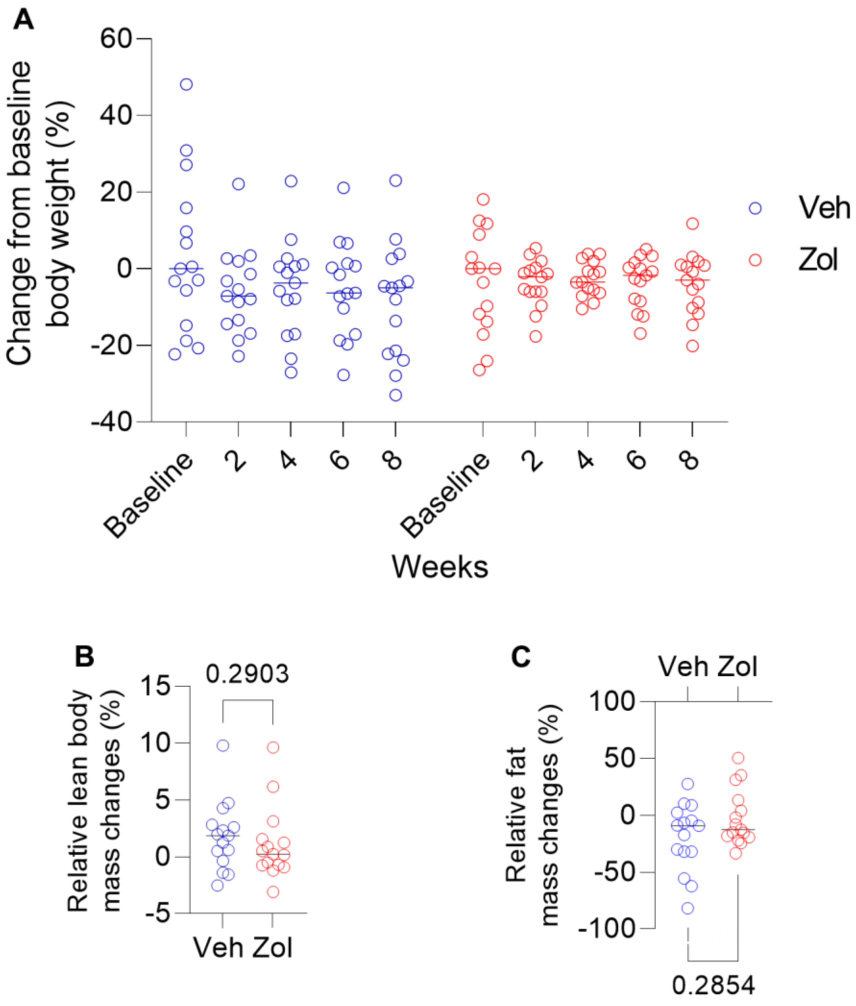

Supplementary Figure 3. Effects of zoledronate on body composition. (**A**) Baseline and weekly percent changes in body weight in the vehicle- and zoledronate-treated mice; percent change over the course of the study in (**B**) lean mass and (**C**) fat mass in the vehicle- and zoledronate-treated mice. *p*-values according to Mann-Whitney test, n=15 mice/group.

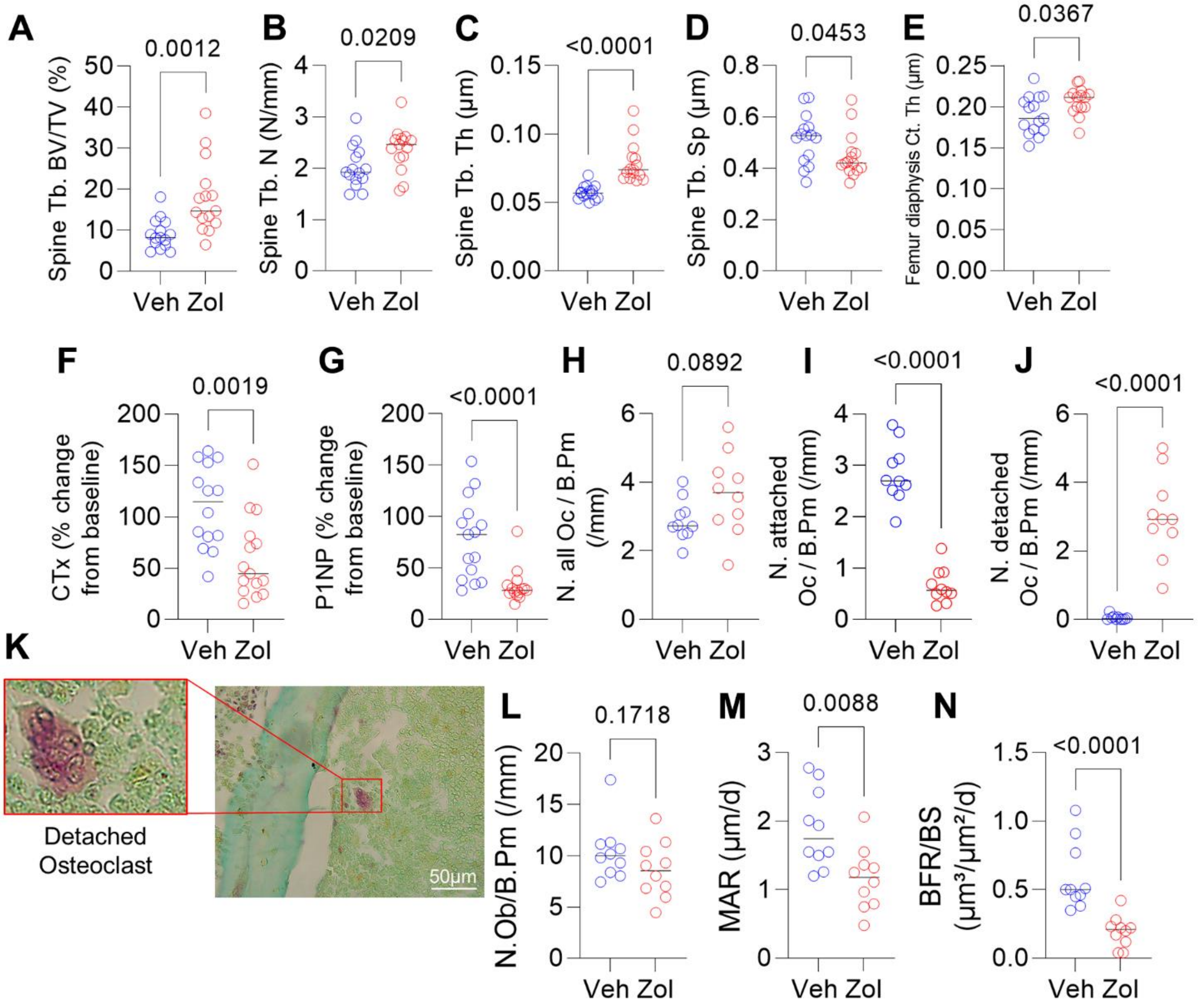

Supplementary Figure 4. Skeletal effects of zoledronate. Effects of zoledronate on (**A**–**D**) spine trabecular and (**E**) femur diaphysis cortical parameters; (**F**, **G**), percent changes in serum CTx and PINP levels in the zoledronate- and vehicle-treated mice; (**H**) total osteoclasts, (**I**) attached osteoclasts, and (**J**) detached osteoclasts in the zoledronate- and vehicle-treated mice; (**K**) shows an example of a detached osteoclast in the zoledronate-treated mice; trabecular (**L**) osteoblast numbers, (**M**) mineral apposition rate, and (**N**) bone formation rate in the vehicle- and zoledronate-treated mice. *p*-values are using Mann-Whitney test; n = 10-15 mice/group.

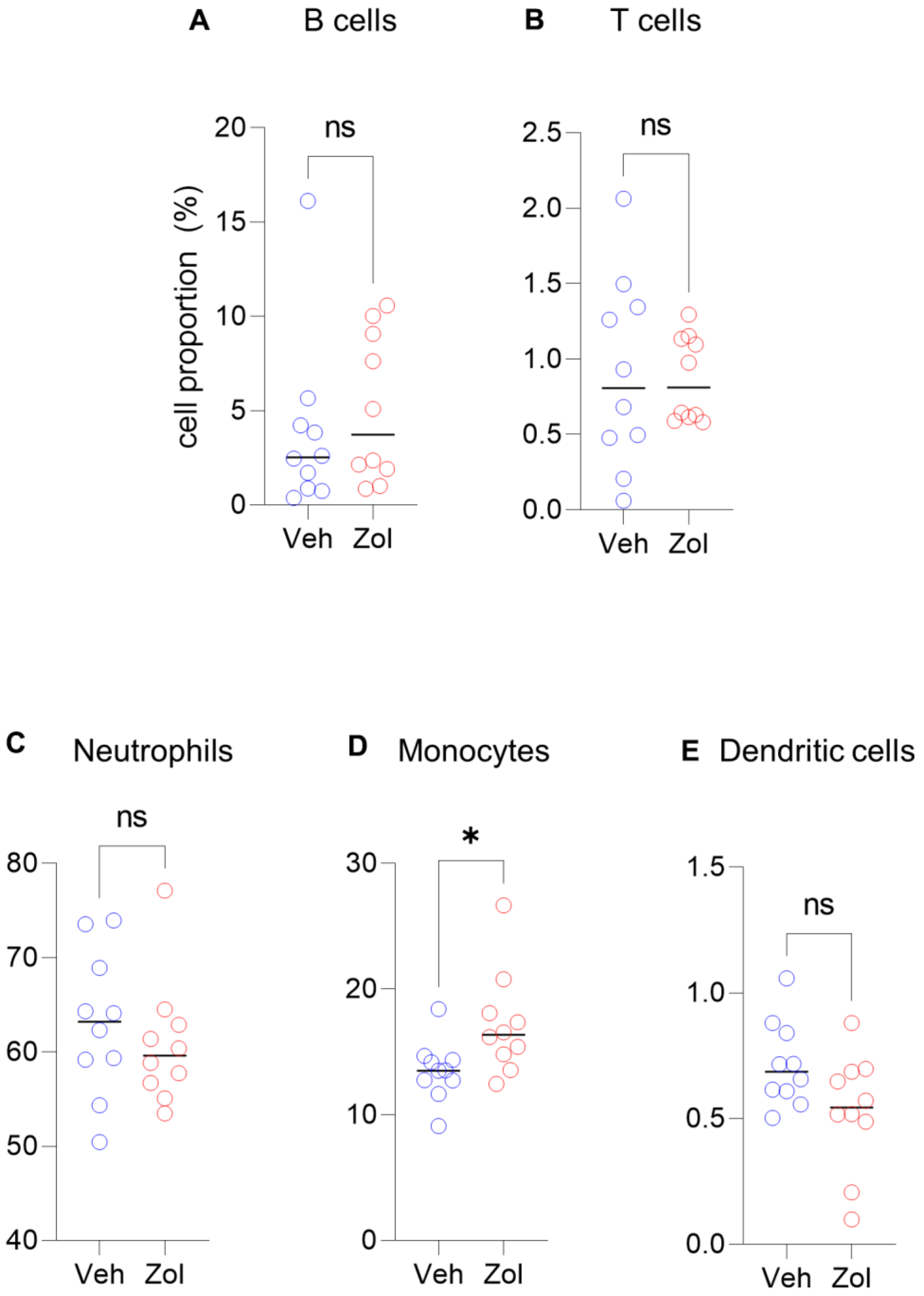

Supplementary Figure 5. Zoledronate does not reduce other bone marrow hematopoietic cell populations. Percentages of (**A**) B-cells, (**B**) T-cells, (**C**) neutrophils, (**D**) monocytes, or (**E**) dendritic cells are not altered by zoledronate. N=10 mice in the control and n=10 in the zoledronate group. Unpaired t-tests, $p<0.05$.
