# Supplementary material for: In vitro and in vivo effects of zoledronic acid on senescence and senescence-associated secretory phenotype markers
Source: Aging (Albany NY). 2023 May 7;15(9):3331–55. doi: 10.18632/aging.204701 (PMC10449299; doi:10.18632/aging.204701)
Supplement: Supplementary Table 1 [file aging-15-204701-s002.pdf]

SUPPLEMENTARY TABLE

Supplementary Table 1. Antibodies used for CyTOF, conjugated metals, supplier and identification numbers.

Reagent	Conjugated metal	Source	Identifier
Hematopoietic			
CD45	089Y	Fluidigm	3089005B
Stem/Progenitor			
CD34	163Dy	Thermo Fisher	MA5-17826
Emcn	164Dy	ThermoFisher	14-5851-82
CD117	173Yb	FDM	3173004B
Granulocytes			
CD11b	111Cd	Biolegend	101201
Macrophage			
F4-80	113Cd	Biolegend	123101
CX3CR1	161Dy	Biolegend	149002
Monocytes			
Ly6C	141Pr	Biolegend	128039
CD11c	142Nd	FDM	3142003B
CCR2	156Gd	Novus	MAB55381-100
CD115	174Yb	Biolegend	135521
CD14	165Ho	Biolegend	123321
T cells			
CD4	145Nd	FDM	3145002B
CD3e	152Sm	FDM	3152004B
CD8a	168Er	FDM	3168003B
Osteoclasts			
Ctsk	147Sm	abcam	ab37259
ACP5	151Eu	Abcam	ab83050
B cells			
CD45R	153Eu	Biolegend	103249
Neutrophils			
Catalase	158Gd	Abcam	ab223793
Ly6G	170Er	Biolegend	127637
B cells			
CD19	166Er	FDM	3166015B
BMSCs			
Ly-6A	169Tm	FDM	3169015B
SASP			
IL-1α	171Yb	Biolegend	503202
IL-1β	159Tb	Cell Signaling	31202
CXCL1	175Lu	R&D Systems	MAB453-500
pNFkB	110Cd	Abcam	ab16502
MCP-1	149Sm	Thermo Fisher	MA5-17040
PAI-1	160Gd	Abcam	ab125687
IL-6	167Er	FDM	3167003B
Senescence			
p53	116Cd	Abcam	ab252388
p16	155Gd	Abcam	ab232402
p21	176Yb	Santa Cruz	sc-6246
DNA Damage			
pATM	114Cd	Invitrogen	14-0146-82
